# Digital Solutions for Community and Primary Health Workers: Lessons From Implementations in Africa

**DOI:** 10.3389/fdgth.2022.876957

**Published:** 2022-06-03

**Authors:** Ayomide Owoyemi, Joanne I. Osuchukwu, Clark Azubuike, Ronald Kelechi Ikpe, Blessing C. Nwachukwu, Cassandra B. Akinde, Grace W. Biokoro, Abisoye B. Ajose, Ezechukwu Ikenna Nwokoma, Nehemiah E. Mfon, Temitope O. Benson, Anthony Ehimare, Daniel Irowa-Omoregie, Seun Olaniran

**Affiliations:** ^1^Department of Biomedical and Health Information Sciences, Chicago, IL, United States; ^2^Mende Primary Health Center, Kosofe Local Government, Lagos, Nigeria; ^3^Social and Behavioral Sciences Department, T.H. Chan School of Public Health, Harvard University, Boston, MA, United States; ^4^Department of Health Informatics, Swansea University, Wales, United Kingdom; ^5^The Neo Child Initiative for Africa, Lagos, Nigeria; ^6^Department of Human and Health Sciences, Northern Illinois University, DeKalb, IL, United States; ^7^Department of Community Health and Primary Care, College of Medicine, University of Lagos, Lagos, Nigeria; ^8^Society for Family Health Nigeria, Abuja, Nigeria; ^9^Department of Obstetrics and Gynecology, National Hospital, Abuja, Nigeria; ^10^Institute for Computational and Data Sciences, University at Buffalo, State University of New York, Albany, NY, United States; ^11^CareMonie Health Financing, Abuja, Nigeria; ^12^Department of Integrated Information Technology, College of Engineering and Computing, University of South Carolina, Columbia, SC, United States

**Keywords:** digital health, mHealth, Africa, primary health, community health, eHealth

## Abstract

The agenda for Universal Health Coverage has driven the exploration of various innovative approaches to expanding health services to the general population. As more African countries have adopted digital health tools as part of the strategic approach to expanding health services, there is a need for defining a standard framework for implementation across board. Therefore, there is a need to review and employ an evidence-based approach to inform managing challenges, adopting best approaches, and implement informed recommendations. We reviewed a variety of digital health tools applied to different health conditions in primary care settings and highlighted the challenges faced, approaches that worked and relevant recommendations. These include limited coverage and network connectivity, lack of technological competence, lack of power supply, limited mobile phone usage and application design challenges. Despite these challenges, this review suggests that mHealth solutions could attain effective usage when healthcare workers receive adequate onsite training, deploying applications designed in an intuitive and easy to understand approach in a manner that fits into the users existing workflows, and involvement of the stakeholders at all levels in the design, planning, and implementation stages of the interventions.

## Introduction

The agenda for Universal Health Coverage has driven the exploration of various innovative approaches to expanding health services to the general population (1). This has coincided with the digital revolution of the 90s' which expanded access to Information globally, and Africa has seen a significant adoption of these services especially in the last two decades. Beyond utilization in communication, finances and transportation, public health and healthcare services have seen greater application of these tools to improve population health (2). From disease outbreak monitoring and surveillance, infectious disease contact tracing, telemedicine services, electronic medical records as well as pharmacogenomic applications for medication prescriptions and machine learning for diagnosis and treatment of certain ailments. These tools have contributed to increased access to health services, improved clinical decision making for clinicians, and increased patient engagement and autonomy (3).

Digital health is a general term used to describe the use of internet solutions, big data, and communications technologies to collect, share and manage health information to improve both individual as well as public health (3). Some of the tools include patient-clinician video conferencing tools, sensors for remote monitoring of physical activities, vital signs including EKGs, blood pressures, blood sugar; drone technology for transporting biological products like blood products, vaccines and lifesaving medications to hard to reach locations, electronic dashboard for tracking disease outbreaks, cloud based-databases for storing and retrieval of patient's clinical records, referrals and electronic directories for locating health facilities (4).

Furthermore, the COVID-19 pandemic exacerbated existing health access disparities in the population from low- and middle-income countries revealing gaps in access to care and worse outcomes especially for people of low socioeconomic background (5). This created a call for innovative approach to strengthening the health system, and digital health technology is one of the tools identified for implementation (6). While there is an increased adoption of digital health tools in Africa, digital technology deployed for health care in some instances are siloed from government response leading to fragmentation of services (2).

However, as these tools are beginning to see widespread adoption and use within the health systems, some challenges arise. Firstly, there is no robust evidence suggesting that the widespread use of digital tools strengthens national health systems or makes health care more accessible or affordable. Furthermore, there are concerns that adoption of digital technologies may reveal underlying systemic health systems challenges of infrastructure and workforce gaps in addition to the local, social, political, and economic contexts (2). Moreover, effective adoption of digital technology in health requires presence of robust digital infrastructure like universal reliable broadband access, widespread smartphone ownership and utilization and affordable data access (7). Furthermore, there is a demonstrable digital divide among the public adoption of digital technology, and these mostly follow patterns along age, low socioeconomic status, literacy rates as well as rural vs urban dwellers (8). Herein presents the challenges with implementation of digital health technology within Africa.

As more African countries have adopted digital health tools as part of the strategic approach to expanding health services, there is a need for defining a standard framework for implementation across board (9). This is more important for primary healthcare in the region as it faces many challenges which include inconsistent quality of health care delivered by professionals, and different limitations in accessing relevant diagnosis and treatment. Digital health has been proposed and applied as a tool in solving some of these challenges (10). However, there is a need to understand what works, how best to implement and manage challenges from previous implementations. This review article assesses implementation of digital health solutions for community and primary healthcare workers across the continent to understand and highlight extant challenges, approaches that worked and useful recommendations for future implementations.

## Methods

In identifying sources for this review, multiple electronic databases were searched. These included MEDLINE *via* OVID, Embase, Web of Science and CINAHL. The comprehensive search was carried out between 10th and 20th of November 2021 by two authors who retrieved articles published in academic journals, using keywords such as “population health,” “primary health care,” “eHealth,” “mHealth,” “digital health” “Africa” and “low resource setting.” The full search strategy is attached in the Supplementary Material. Abstracts and full-text articles were searched manually in digital sources. Reference harvesting and individual searches for author names were further carried out to identify additional relevant articles. No time limits were applied.

Two authors independently reviewed the abstracts and included original research articles using specified inclusion criteria: original articles written in English, research work related to Africa, literature focused on public/community/population health initiatives and literature focused on health workers in the above contexts. The exclusion criteria were reviews, unpublished manuscripts and research work that focused on clinical use cases without the involvement of community and primary health workers.

An extraction form was prepared and data that was relevant to the study objectives and guided by a recent similar review by Addotey Delove et al. included: author, country, purpose of the study, type of mHealth intervention, health condition, population, type of study, challenges faced, management of these challenges, successes and recommendations made for future implementations and other notes (11).

## Results

The original search returned 9,030 articles. Out of these potentially relevant articles, 1,120 studies were excluded because of duplication using a reference manager. Screening the title and abstract of the remaining records for eligibility allowed for the exclusion of further 7,839 records.

This led to a further detailed review of 71 articles for which full-text articles were sought and then reviewed by the authors. Research methods used in the included studies included qualitative studies, surveys, mixed methods, cross sectional studies and cohort studies. The papers reported different applications of mHealth to different health issues and conditions which included maternal and child health (44 studies), infectious diseases (16 studies), health management and cardiovascular health (six studies), ear health and cervical cancer (two studies), and mental health, eye care, logistics (one study each). 15 of the included studies were done in Kenya, 13 in South Africa, 11 in Ghana, 10 in Malawi, six in Uganda, four in Nigeria and Zambia, three in Ethiopia, three in Tanzania, two in Sierra Leone, one in Botswana, Lesotho, and Rwanda.

### Challenges

#### Limited Coverage and Network Connectivity

Lack of reliable wireless data availability, coverage, and network access disrupted the implementation process. Some sites experienced wireless network downgrades to a slower data network which disrupted data downloads and uploads to the server, others were limited in their ability to use applications due to airtime, network and connectivity issues (12–15). Although consultation with providers and the onsite technical team were ongoing to improve coverage, a good number of participants still reported difficulty with site access (13, 16). A potential roadblock in the future will be the difficulty in implementing mHealth technology in remote areas due to ongoing mobile network connectivity issues and poor network penetration (12, 17–22).

Another issue identified was server connectivity problems where the mHealth tool was unable to connect with the server while other programs ran simultaneously (16). Additionally, there was delayed reporting and variation in data collection due to network accessibility and connectivity; certain applications had to work offline pending network availability. Timely reporting became dependent on network connectivity which defeats the purpose of the use of mobile health technology to improve health services (13, 17, 19, 23–25).

#### Inadequate Technological Competence

Studies have shown that the introduction of a new device requires adequate training on the device to ensure technological competence. The consistency and frequency in training will likely impact the confidence and competence level of users of the devices; in both patients and providers (26). Users were unable to utilize mobile phones and web documents as required due to capacity limitations and this led to irrelevant and inaccurate retrieval of information (27, 28).

Literacy in digital communication in low-resource settings is key to boosting the relationship between providers and patients. This link may help to increase comprehension and treatment compliance, thus being innovative in the development of mHealth systems for digital communications in rural areas is imperative (27).

#### Poor Power Supply

While studies emphasize the potential impact of mHealth services with the use of mobile phones to boost information systems and decision-making within the community, several hurdles affecting digital health solutions implementation were also highlighted. One of the key challenges includes sporadic power supply and lack of power outlets (7, 9–11, 14–16, 18, 29, 30). This impacted the ability to power devices, use applications on mobile phones, central database systems interruption, and server downtime during power interruptions (20, 23, 31, 32). Another notable impact was variation in data reporting, delayed responses, and duplicative work (24, 32).

#### Product Design Challenges

Product design challenges address the issues faced by users of the mHealth system and recommendation for future implementations of comparable mHealth systems. These issues ranged from application crashes, data retrieval restrictions, screen freezing leading to login restrictions, submission errors, and improper integration with the existing electronic health information system of the facility (33–36).

### Management of Challenges

The challenge of low digital literacy among healthcare workers was mediated through adequate training of staff including onsite mentorship, provision of equipment and infrastructure (17, 20, 23, 25, 33, 37–40). Routine technical support troubleshooting and fixing both software related problems (related to applications) and hardware related problems (related to phones and sim cards) were provided by the local research team including installing the phone system applications, protocol forms and dealing with any queries (12, 13, 17, 21, 27, 41). A troubleshooting guide was developed, and training was provided for supervisors and health sector health information technicians and other program staff. These health workers were able to address most of the common problems using the troubleshooting guide and with remote support from the regional and central teams. For instance, in Ethiopia the mHealth technology to support service provision and strengthen linkages within the primary health care units (PHCUs) provided *ad hoc* and routine onsite support and supervision to end-users (33).

Studies conducted in Zambia, Tanzania, Uganda, Nigeria and Ethiopia reported use of solar lamps/chargers and power banks to recharge the smart phones (2, 3, 7, 8, 23, 26). In some other settings like Kenya, community health workers used paper to record values, and later sent them when power was restored (24, 31). In some studies, it was reported that there were insurgent attacks and cases of phone theft. A research study to improve delivery, timeliness and quality of maternal and child health services by leveraging existing mHealth technology to support service provision reported that from a total of 308 smartphones distributed to mHealth users, 7(3%) tablets were reportedly lost and 86(28%) were damaged within the first year of implementation (8) (33). To ensure security, a military convoy accompanied the health workers on field trips. In addition, phones were tested before deployment with spare batteries and power banks for charging. For cases of stolen phones, they were immediately replaced, and users and staff were encouraged to become temporary owners of the phones as ownership was a strong motivator for the health workers, who recognized the value and usefulness of the devices, so took care to look after them (13, 19, 20, 42).

Poor network connectivity was another common challenge which was managed by using alternative network providers or use of design changes to the user interface that had an offline functionality design to ensure operation during network downtime and the ability to save forms for later (13, 20, 23, 27, 31, 40, 43–46).

### Approaches That Worked

#### Background Research

Detailed background research provided first-hand information about the problem—such as an illness—to be tackled in the community as well as how best to handle it (47). Furthermore, the implementers of these user-centered digital initiatives ensured to acquire as much information about the community health workers, location, need, opportunities, and other information relevant to the implementation and acceptability of the solution (46, 48, 49). These included pre and post evaluation of the knowledge of health care workers and feedback from test users (50, 51). The questions addressed topics about the design and usability of the application as well as pertinent issues concerning potential blockades to its mass adoption (48, 52, 53). They sought to gain knowledge from the successes and failures of past implementers. For example, implementers of LIFE (Life Saving Instruction for Emergencies) tried to understand the learning processes of clinicians using their digital application (54). This consequently signified the need to deliver more tailored training. Also, exploring the interviews in the comfort zones of the interviewees ensured more honest feedback (55).

#### Stakeholder Involvement

Involvement of the stakeholders at all levels in the app design, planning, and implementation has been instrumental in the successful adoption of the initiatives. This was true for inputs from health care workers at the grassroots level up to the support obtained from governmental organizations at the top (51, 54, 56, 57). Empowered health care workers connected with the initiatives better when they saw themselves as co-owners (42). They ensured the solutions were locally relevant and worked hard to see to successful training sessions (39). The LIFE smartphone application which has over 5,000 users in Kenya secured partnerships with stakeholders such as the Kenyan Pediatric Association and the Nursing Council (54). This has enabled them to offer Continuous Professional Development points to users who complete the training in due time. Collaboration with stakeholders also provided important avenues for feedback which helped optimize chances of success (35, 58).

#### Features for Increased Productivity

The successful applications introduced features that increased the efficiency and accuracy of clinic activities within the respective locations. Standardized data collection tools, reliable health surveillance networks, and smart diagnostic algorithms provided data that enabled improved clinical decision making (22, 27, 50, 59–62). In contrast to paper-based data collection tools, this reduced the incidence of missed and wrong diagnoses, thus enabling appropriate treatments or referrals to higher cadres of health care when necessary (22, 44). It also facilitated quality storage and security of healthcare data (50, 54). The community health workers were satisfied with the increased efficiency and reduced burnout (22, 25). Community health workers, who integrated mHealth applications in clinical duties, managed patients just as accurately and in degrees comparable to trained professionals (36, 63).

Furthermore, the real-time data transfer and improved communication channels among health care providers in the various cadres of management, as well as with the patients, encouraged continuity of care, reduced clinic waiting times, and improved quality of patient care (17, 46, 62). The opportunity to compare reports detailing facility activities gave way for stricter quality assurance measures (46). Additional benefits such as health education and check-up reminders *via* SMS further provided opportunities for caregivers to advocate for healthy habits and preventive behaviors (25, 40, 45, 64).

#### Ease of Use

The user-centered platforms were designed to be as easy to understand as possible; not requiring high technical skills for usage (13, 23, 50, 60). Leveraging existing paper-based frameworks, applications that provided digital forms were intuitive and undemanding (21, 65). Users liked when it suggested next steps because this removed the responsibility of having to memorize every detail. On occasions, developers had taken measures to add features that further simplified the experience. This can be seen with the Family Planning Mobile Job Aid application which has an inbuilt regional language and voice-based response system that reflects population diversity (54). This, as well as other perceived beneficial features, resulted in high acceptability among targeted community health workers. In addition, initiatives that had flexible implementation protocols also had high acceptance rates (12, 56, 66).

#### Training Sessions

Training sessions before deployment, when offered, have been shown to increase knowledge and the participation of community health workers (23, 34, 37, 59, 67). This had sometimes been conducted *via* community fora or classroom trainings (67, 68). Doukani et al. (41) however, mentioned the importance of holding the training in a health center as it allowed for easier interaction and troubleshooting while the participants used the application. Health Surveillance Assistants at Malawi stated that using the application improved their understanding and subsequent interactions with patients who now had more trust in the accuracy of their treatments (65).

#### Leveraging Existing Frameworks

To thrive, the applications were designed in ways that were intuitive and easy to understand. Oftentimes, this involved adapting the details of already existing and approved programs or paper-based forms (33, 51, 56, 58, 65). For example, cStock, an mHealth logistics tool in Malawi, integrated mobile-based tools with existing health management systems (18). Also in West Nile, Uganda, a pilot mHealth intervention designed to increase coverage of Intermittent Preventive Treatment of Malaria in pregnancy, integrated an SMS platform into an already existing platform (68). Sometimes, the integration encompassed leveraging on the services of the community health workers, themselves, in rural settings (31). It was also necessary to ensure that these digital initiatives were in line with existing policies and tailored to fit local contexts (23, 33, 51, 56).

#### Others

Privacy was achieved by ensuring that cell phones used were provided by the study and were password protected (13, 41). A sense of anonymity was maintained by concealing personal information and avoiding face-to-face interactions. The cost of implementation was significantly reduced by integrating open-source software for the application design and deployment (23, 31). This also helped to maintain sustainability. Additionally, during times of limited internet connection, data gets stored within an application until the connection gets restored (69). A study introduced real-time monitoring of health worker activities through a cloud system to ensure quality and create opportunities for intervention when required (70).

### Recommendations for Future Implementations

The following recommendations were made for mHealth implementation in Africa:

Health care workers need to be adequately and continuously trained and empowered on the use of these technologies (24, 39, 46, 49, 53, 56, 63, 71, 72). There is a need to consider the integration of interventions into existing workflows alongside regular training, case management guidelines, emergency supplies, equipment, and drugs, this should also include taking into account the possibility of other ongoing interventions in the system (47, 73). Measuring and establishing the acceptability of the mobile health interventions by health care workers is very critical in the sustainability and scalability of any mHealth pilot project (74). Researchers recommended an incremental scale-up with stronger mentoring and supervision as well as evaluation of the feasibility, effectiveness, and cost-effectiveness of implementations (75, 76).

Routine technical support is required to address the technical challenges community health workers face in their day-to-day interaction with the application on their mobile phones. This can be overcome by empowering members of staff with the skills to troubleshoot the functionalities of the mobile applications (17). Staff need frequent feedback on how routine practices are being performed (mainly diagnostics and case management); but also, on the utility of data they collect to understand the importance of good quality data for improving health (58).

There is a need for better evaluation and high-quality trials to establish the effects of clinical diagnosis and management support such as protocols and decision support systems on clinical outcomes using mobile devices (19, 23, 42). They also advocated focused group discussions with the study participants during the pre-implementation phase to shed light on challenges and guide the team better. Researchers have also suggested that additional research to reassess the impact of mHealth interventions on organizational structures and warned against an over reliance on technology to solve complex human resource health problems (77).

Implementing mHealth requires larger investments of time and resources for supervision and development of the needed applications (25). It is important to address human resource issues, and level of financial reimbursement for the designated roles. Project implementation managers should improve monitoring and evaluation using stronger technical oversight (22). Usability studies should be conducted to ensure that individual, context and systemic factors are understood to help in subsequent implementation (78). Instead of outsiders, existing in-house data managers and coordinators should be consulted for the tasks, thereby emphasizing the importance of having “a pool within the system of who can train new one, training of trainers” to build capacity and ensure longer-term sustainability (36).

Key stakeholders, partners, and the government need to streamline data input requirements and enhance automated generation of required government reports on number of deliveries and other key health indicators (21, 24, 39). Applications should be more in line with the existing eHealth framework to ensure that all projects follow standard practice and are aligned with specific interests, and the local administration should lead the wider implementation for program to continue to be successful (36). Capacity building is required to ensure policy makers at all levels could use and interpret health data, while it is also important that health system staff understand the significance of data for local program management (58). Lastly, it is important that tools developed elsewhere should be adapted to the local context before it is deployed (79).

## Discussion

This review identified public health digital interventions executed in low- and middle-income countries, the implementation approaches, challenges encountered, and made recommendations for future projects. When designed and implemented to meet user needs, the use of mobile health applications could serve as cost effective tools to considerably enhance the administration of public health projects, support community health workers and improve patient outcomes when used as tools for education, disease/compliance monitoring, and for information acquisition (80–82). However, low- and medium-income countries are challenged by unique economic and social disparities such as literacy, lack of infrastructure and effective government policies that pose barriers to implementation, access, and effective utilization of mHealth platforms by patients and healthcare workers (36, 80, 83).

Evidence from the reviewed papers showed that limited internet coverage and poor network connectivity is one of the major barriers facing successful implementation of mHealth innovations in Africa, this is similar to studies conducted in other low and middle income (LMIC) areas like China and India (12, 13, 17, 84, 85). Other barriers such as unavailability, poor accessibility and unreliable power supply are direct symptoms of poor infrastructure and ICT development (20, 22–24, 31, 32, 66, 71). Some of these problems are not peculiar to Africa as they have been reported in Asian and South American countries (86). The development of relevant infrastructure and other ICT systems which are the backbone of mHealth innovations must be addressed to achieve success in the deployment of mHealth innovations (31). Though implementers have devised various means to overcome these barriers such as provision of alternative network providers, using design elements with offline functionalities, provision of solar powered lamps and phone chargers, manual data recording during power outages, etc., these are all temporary solutions which will increase the cost of implementations (13, 20, 23, 31, 43). The overall success of mHealth implementation across Africa will depend on the adequate investment in ICT infrastructure development which will create an environment that will provide the availability and reliability of the internet and electricity supply which will in turn ensure subsequent successful implementation of mHealth innovations.

Digital illiteracy is another barrier that impairs successful implementation of mHealth innovation in Africa, this problem has also been found in implementations in other LMICs (27, 87). These challenges are more evident in the rural than urban regions. According to Kallander et al., some of the major considerations for successful implementations are collaboration, literacy, culutre and partnerships (88). Improvement in digital literacy specifically in rural regions and generally across Africa can be achieved by bringing together major stakeholders *via* multisectoral and multidisciplinary collaborations. These stakeholders could create training and mentorship programs and ensure provision from both government and non-governmental organizations contributions to special funding pools for mHealth projects to boost the uptake of mHealth innovations and improve the efficiency of mHealth implementations (20, 23, 25, 33, 37–40, 84). Lastly, to address product design challenges causing technical difficulties during implementations, developers should ensure that mHealth applications could be integrated with the current facility-level electronic health information system while they empower the local team to be able to provide routine technical support in order to create a self-sustaining mHealth system model (35).

## Conclusion

This review highlights key challenges inherent in implementation of digital health solutions in low-and-medium income countries in sub-Saharan Africa. These include limited coverage and network connectivity, lack of technological competence, lack of power supply, limited mobile phone usage and application design challenges. Despite these challenges, this review suggests that mHealth solutions could attain effective usage when healthcare workers receive adequate onsite training, deploying applications designed in an intuitive and easy to understand approach in a manner that fits into the users existing workflows, and involvement of the stakeholders at all levels in the design, planning, and implementation stages of the interventions. Although there is need for further research to establish the direct impact of mHealth tools on clinical outcomes and to generate best practice frameworks for implementation, this paper reveals the importance of larger investments of time, resources, and policies to support the development of infrastructures that can enhance the uptake and effectiveness of digital healthcare solutions in sub-Saharan Africa.

This study is limited by the exclusion of studies not done in English language, which would have limited the number and scope of studies found. Also, most resources were from the peer-reviewed literature, which would lead to the exclusion of a lot of other mHealth implementations that might exist in reports and other gray literature resources.

## Author Contributions

AO, JO, and CAz contributed to all aspects of the article. RI conducted the search with AO and edited different aspects of the article. BN, CAk, GB, AA, EN, TB, AE, DI-O, and SO worked on the knowledge synthesis and contributed to editing and proofreading the article. AE, DI-O, and SO worked on the discussion and conclusion aspects of the article. All authors contributed to the article and approved the submitted version.

## Conflict of Interest

JO is presently employed by the Kosofe Local Government, Lagos, Nigeria. CAk is presently employed by The Neo Child Initiative, Nigeria. DI-O is presently employed by CareMonie Health Financing, Nigeria. The remaining authors declare that the research was conducted in the absence of any commercial or financial relationships that could be construed as a potential conflict of interest.

## Publisher's Note

All claims expressed in this article are solely those of the authors and do not necessarily represent those of their affiliated organizations, or those of the publisher, the editors and the reviewers. Any product that may be evaluated in this article, or claim that may be made by its manufacturer, is not guaranteed or endorsed by the publisher.
