# Supplementary material for: Digital Solutions for Community and Primary Health Workers: Lessons From Implementations in Africa
Source: Front Digit Health. 2022 Jun 3;4:876957. doi: 10.3389/fdgth.2022.876957 (PMC9215204; doi:10.3389/fdgth.2022.876957)
Supplement: Supplementary file 1 [file Table_1.DOCX]

**Search Strategy**

| **Population** |  | **Concept** |  | **Concept** |
| --- | --- | --- | --- | --- |
| *** Africa * OR OR “Developing Countr*” OR “Low and Middle Income Countr*” OR “Low resource settings”** | **A**  **N**  **D** | **“digital solution*” OR “digital health” ORehealth OR mhealth OR telehealth** | **A**  **N**  **D** | **“Public health”OR “Community Health” OR “Primary healthcare” OR “population health”** |
